# Overexpression of a New Osmotin-Like Protein Gene (*SindOLP*) Confers Tolerance against Biotic and Abiotic Stresses in Sesame

**DOI:** 10.3389/fpls.2017.00410

**Published:** 2017-03-28

**Authors:** Supriyo Chowdhury, Arpita Basu, Surekha Kundu

**Affiliations:** Molecular and Applied Mycology and Plant Pathology Laboratory, Department of Botany, University of CalcuttaKolkata, India

**Keywords:** defense signaling, osmotin-like protein, charcoal rot, drought, salinity, over-expression, sesame, GFP

## Abstract

Osmotin-like proteins (OLPs), of PR-5 family, mediate defense against abiotic, and biotic stresses in plants. Overexpression in sesame of an OLP gene (*SindOLP*), enhanced tolerance against drought, salinity, oxidative stress, and the charcoal rot pathogen. SindOLP was expressed in all parts and localized to the cytosol. The transgenic plants recovered after prolonged drought and salinity stress, showing less electrolyte leakage, more water content, longer roots, and smaller stomatal aperture compared to control plants. There was an increase in osmolytes, ROS-scavenging enzymes, chlorophyll content, proline, secondary metabolites, and reduced lipid peroxidation in the transgenic sesame under multiple stresses. The OLP gene imparted increased tolerance through the increased expression of three genes coding for ROS scavenging enzymes and five defense-related marker genes functioning in the JA/ET and SA pathways, namely *Si-Apetala2, Si-Ethylene-responsive factor, Si-Defensin, Si-Chitinase*, and *Si-Thaumatin-like protein* were monitored. The transgenic lines showed greater survival under different stresses compared to control through the integrated activation of multiple components of the defense signaling cascade. This is the first report of transgenic sesame and first of any study done on defense-related genes in sesame. This is also the first attempt at understanding the molecular mechanism underlying multi-stress tolerance imparted by an OLP.

## Introduction

Combinations of abiotic and biotic stresses, instead of individual ones, create a realistic threat to crop cultivation throughout the world. The concurrent presence of an abiotic stress either aggravates or impedes the effect of infection by a pathogen, thereby rendering a plant susceptible or tolerant against the combined stresses (Ramegowda and Senthil-Kumar, 2015). This is particularly important in case of the important oil/seed crop sesame which flower during the dry hot season when drought-induced reduction in cellular water-potential makes the plants more vulnerable to charcoal rot disease by *Macrophomina phaseolina* (Chowdhury et al., 2014a). Although, India is the largest producer of sesame in world producing 0.63 million tons/year, there is about 50% loss in yield due to the different biotic and abiotic stresses (Deepthi et al., 2014).

Recent studies pointed out that under combined abiotic and biotic stresses, early defense signaling events in plants are likely operated by phytohormones but however the modulation of stress signal, crosstalk, and subsequent downstream events are later tailored for specific stress responses (Ramegowda and Senthil-Kumar, 2015). Stress signaling in plants in response to abiotic and biotic factors can induce separate and overlapping sets of genes, leading to the expression of distinct as well as common components (Zhu et al., 2014; Mellacheruvu et al., 2016). These separate pathways show nodal points where they converge and cross-talk to optimize the various defense responses (Xiao et al., 2013), resulting in shared stress mitigation strategy by combined morpho-physiological processes as well as by molecular responses (Pandey et al., 2015). Identification of cross talks between abiotic and biotic signaling pathways has been crucial for envisaging and strengthening our understanding of regulation of plants response against combined stresses. Genes such as those coding for Osmotins or Osmotin-like proteins that impart abiotic as well as biotic stress tolerance therefore present an opportunity to study the mechanism underlying tolerance to dual stresses.

Osmotin-like proteins (OLPs), that resemble Osmotin, a group of 24–26 kDa proteins belonging to the PR-5 group, was originally isolated from tobacco cells under osmotic stress (Choi et al., 2013). Under drought or salinity stresses, osmotin/osmotin like proteins (OLPs) maintains cellular osmolarity by compartmentalization of solutes or by structural and metabolic alterations. Recently we have cloned and characterized a new OLP gene from *Solanum nigrum* (*SindOLP*, BankIt 1588324 Solanum KC292261), which exhibited *in-vitro* antifungal activity against *M. phaseolina* (Chowdhury et al., 2015). As reported in previous studies, the synthesis and accumulation of OLPs depend on osmotic conditions, signifying its involvement in osmotic adaptations (Patade et al., 2013; Weber et al., 2014; Chowdhury et al., 2015). On the other hand a few reports have shown that osmotins and OLPs enhance tolerance to biotic stresses (Subramanyam et al., 2012; Vasavirama and Kirti, 2012; Choi et al., 2013), although the underlying biological mechanism remains unexplored. None of these studies showed that a single OLP gene can confer resistance to abiotic as well as biotic stresses in the same plant, leading to multi-stress resistance. Moreover, no prior studies investigated how the overexpression of an OLP gene affects the regulation of other defense-related genes in plants. The effect of OLP transgene on the regulation of marker genes functioning in the different signal transduction pathways leading to multiple stress tolerance therefore remains largely unknown.

Most of the studies on multi-stress resistance have been done on model plants. Studies on multi-stress resistance in other plants are few and even fewer studies explore the defense signaling mechanism(s) underlying multi-stress resistance in economically important plants (Zhu et al., 2014). In spite of the importance of sesame as an oil and seed crop, development of transgenic sesame was not feasible until recently due to the unavailability of a transformation protocol. The main obstacle was the severe recalcitrancy *in-vitro* that was overcome in our laboratory by establishment of a high frequency transformation protocol for sesame (Chowdhury et al., 2014b). Moreover, there is no study thus far about any gene regulation in sesame in response to any kind of stress. The recent availability of the sesame genome sequence (Zhang et al., 2013) is likely to change that scenario.

Here we overexpressed the new *SindOLP* in sesame not only to study its potentiality in conferring abiotic and biotic stress resistance but more importantly to get and insight into the mechanism by which the stress signaling takes place in these transgenic plants. The subsequent metabolic and molecular changes in response to biotic and abiotic stresses were observed in these transformed lines. The ability of the plants to recover after stress conditions and the expression of genes related to ROS scavenging system were studied. Five defense-related marker genes functioning in the JA/ET and SA signaling arms of defense response to multiple stresses were monitored in the transgenic plants to get an insight into the role of *SindOLP* in multi-stress tolerance. To the best of our knowledge, this is the first report of transgenic sesame. Moreover, this is also the first study of defense-related genes in sesame and the first attempt to analyze the molecular mechanism underlying enhancement of multi-stress tolerance by an OLP.

## Materials and methods

### Cloning of full length *SindOLP* into binary vector

**Figure 1:**
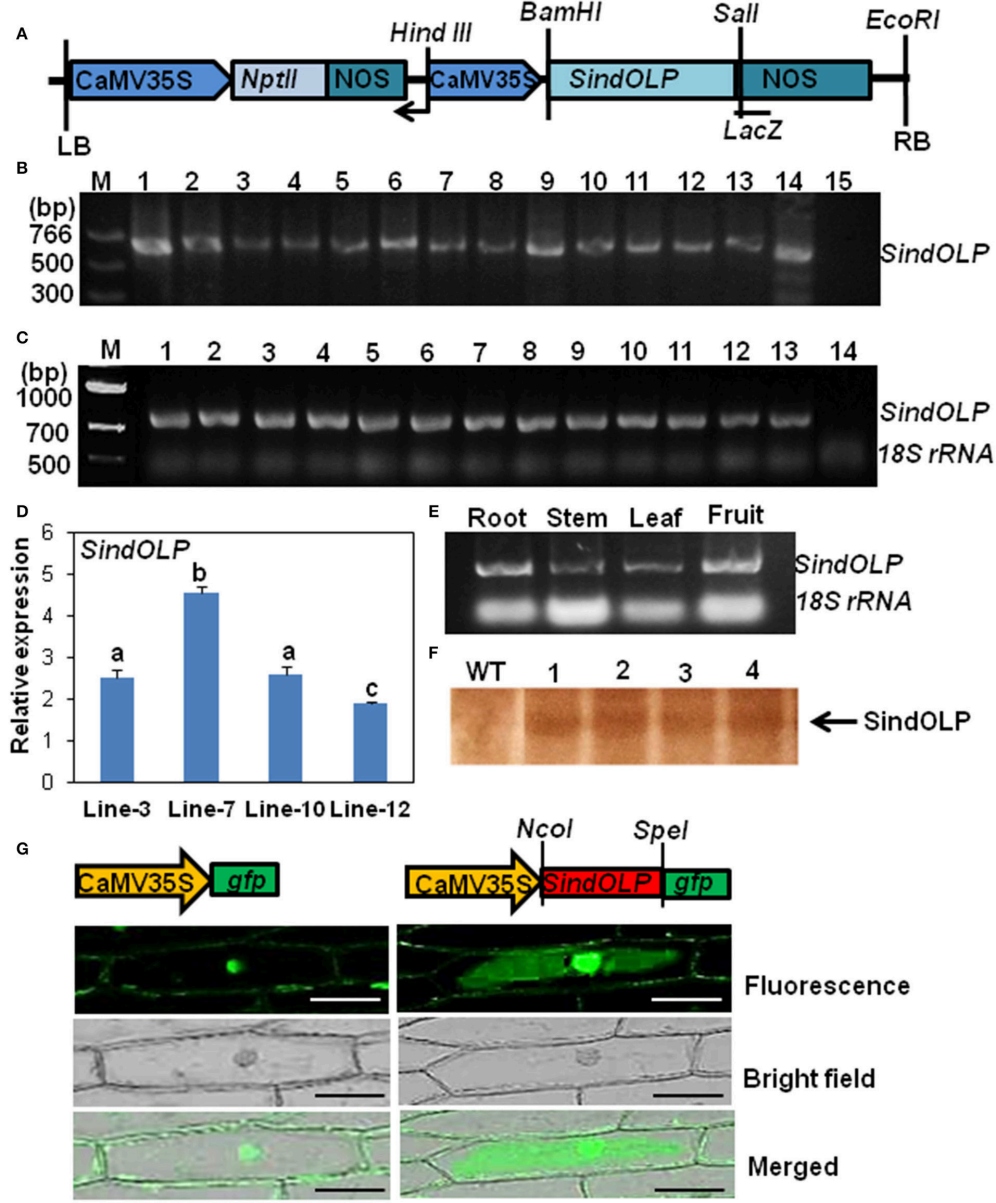
**Molecular characterization of transformed sesame plants carrying *SindOLP* sequence. (A)** Schematic representation of the T-DNA region of the construct pZPY112: *SindOLP* used for sesame transformation. NOS = nopaline synthesis terminator, NptII = Neomycin phosphotransferase II (Kanamycin resistance) gene. **(B)** PCR analysis of genomic DNA from different transgenic lines using *SindOLP* specific primers. M = molecular weight marker, 1–13 = transgenic lines, 14 = pZpy112: *SindOLP* plasmid (positive control), 15 = WT sesame (negative control). **(C)** Expression of *SindOLP* in transgenic lines analyzed by semi-quantitative RT-PCR. 1–13 = transgenic lines, 14 = non-transgenic plants, *18S rRNA* was kept as loading control. **(D)** Relative levels of *SindOLP* transcripts in four transgenic lines by q-RT-PCR. Bars represent mean ± S.E.M of three independent experiments with three replicates. Different letters above bars represent significant difference at *P* < 0.05. **(E)** Expression of *SindOLP* in different tissues of transgenic Line-7. **(F)** Immunoblot analysis of transgenic lines by anti-SindOLP antibody, 1 = line-3, 2 = line-7, 3 = line-10, 4 = line-12. **(G)** Subcellular localization of GFP-tagged SindOLP in onion epidermal cells (Bar = 200 μm).

Cloning of *SindOLP* from genomic DNA of *S. nigrum* L. in pBSKS (Stratagene) vector was done according to our earlier published report (Chowdhury et al., 2015). It was sub-cloned under CaMV35S promoter at the BamH1/Sal1 site of the plant binary vector pZPY112 (Figure 1A). The pZPY112::*SindOLP* was used to transform *Agrobacterium tumefaciens* LBA4404 strain by freeze-thaw method (Chen et al., 1994) and used for sesame transformation.

### Transformation of sesame

The pZPY112::*SindOLP* was used for transformation of sesame (*S. indicum* cv.VRI-1) according to our earlier report (Chowdhury et al., 2014b). The transformants were regenerated on selection medium containing 50 mgL^−1^ Kanamycin and 500 mgL^−1^ Cefotaxime. A total of 13 independent transformants were obtained by preliminary screening in Kanamycin (50 mg/L) and PCR screening using *SindOLP* primers (Figure 1B). Selected plantlets were transferred onto rooting medium (MS basal salts with 3% sucrose, 4.57 μM IAA and 10 mgL^−1^ Kanamycin). Transformants were sub-cultured three times onto fresh regeneration medium containing Kanamycin. After rooting, shoots were transferred into pots filled with soil-rite (soil substitute, Keltech, India). The primary transgenic lines (T_0_) were self-pollinated and their seeds (T_1_) were germinated in MS. Leaves from different T1 individuals from common parental T0 line were checked for kanamycin resistance and data were analyzed using Chi-square (χ^2^) test as described earlier (Chowdhury et al., 2014b).

### Transformation of *Macrophomina phaseolina* with GFP construct

The charcoal rot pathogen *M. phaseolina* was transformed using binary vector pCambia 1302 (Supplementary Figure S8) using our previously published protocol (Basu et al., 2016).

### Detection of copy number of *OLP* of transgenic sesame lines by q-RT-PCR

Transformed lines were screened for *SindOLP* gene sequence by PCR. The full length ORF of 730 bp *SindOLP* was amplified with primers SindOLP (f)-SindOLP(r) (Supplementary Table S4) using PCR conditions as published before (Chowdhury et al., 2015) and sequenced (Supplementary Figure S1B). Transgene copy number of *SindOLP* in the T0 and T1 lines were calculated by qRT-PCR using comparative C_*T*_ method. The sesame Oleosin gene (GenBank AF302807.1), a single copy gene in the sesame genome (Tai et al., 2002), was used as endogenous control for copy number calculation according to standard protocol (Weber et al., 2014; Chowdhury et al., 2014b).

### Protein analysis by SDS-PAGE

Total soluble protein was isolated from WT and transgenic plants according to Mandal et al. (2002). Fifty micrograms protein was loaded in each lane of a 12% SDS-PAGE and separated using a Mini-Protean II electrophoresis Cell (Bio-Rad laboratories, Hercules, CA).

### Detection of SindOLP in transgenic sesame lines by immunoblot

His-tagged SindOLP, purified by Ni-NTA affinity chromatography (Chowdhury et al., 2015) was used to raise anti-*SindOLP* antisera in rabbit. For immunoblot analysis, 50 μg of protein from WT and transgenic lines were separated by 12% SDS-PAGE and transferred onto PVDF membrane using Mini-Trans-Blot (Bio-Rad Laboratories, Hercules, CA). Immunoblotting was done using rabbit anti-*SindOLP* polyclonal antisera (1:200 dilution) and goat anti-rabbit horseradish peroxidase conjugated antibody (1:500 dilution; Genei, India).

### Subcellular localization of *SindOLp*

**Figure 2:**
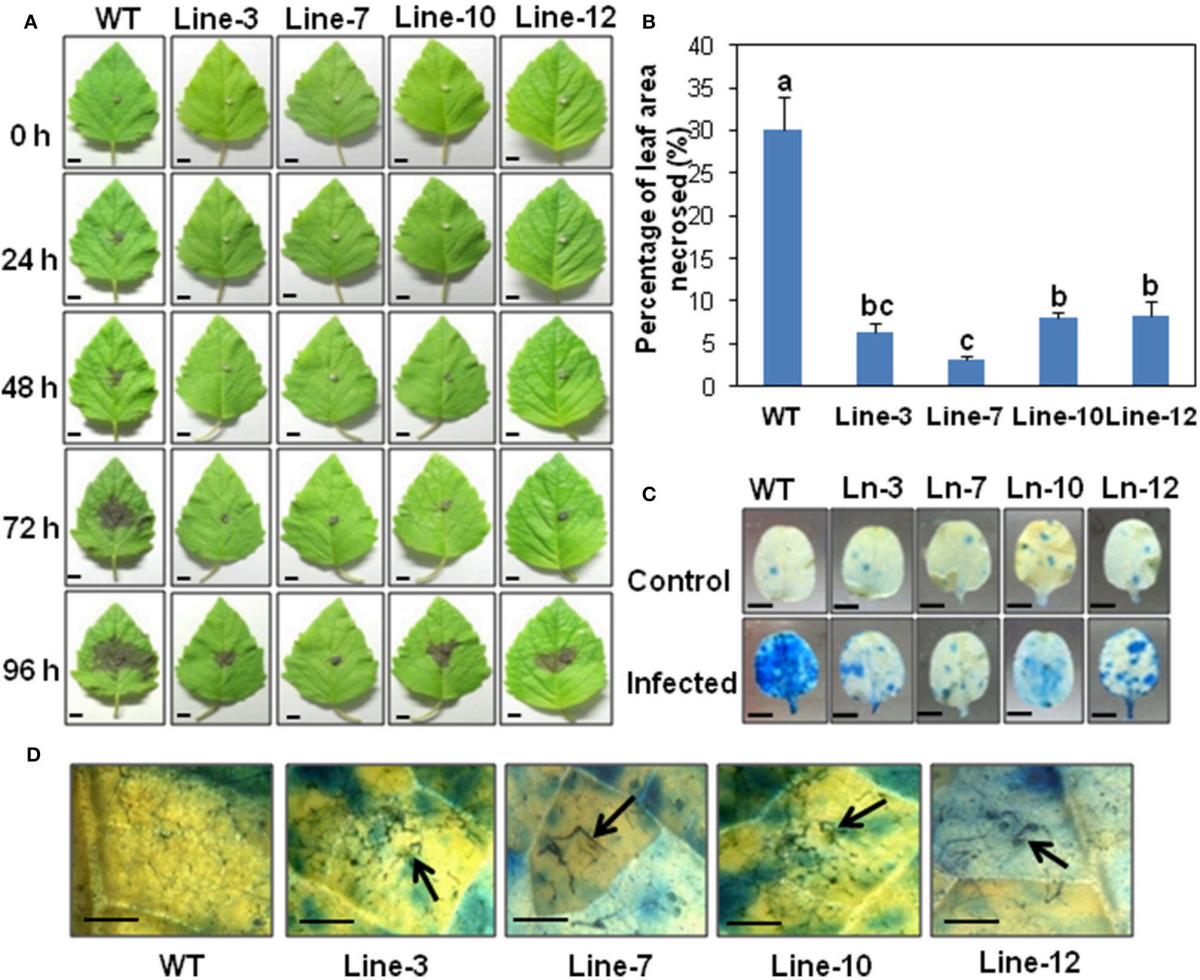
**Detached leaf assay showing enhanced resistance against *Macrophomina phaseolina* in sesame overexpressing *SindOLP*. (A)** Detached leaves from the WT and transgenic lines, inoculated with mycelial disc of *M. phaseolina*, showing development of necrotic lesions at different time points (bar = 1 cm). **(B)** Graph showing percentage of leaf area necrosed after infection in WT and transgenic lines. Bars represent mean ± S.E.M of three independent experiments with three replicates. Different letters above bars represent significant difference from WT (*P* < *0.05*). **(C)** Symptoms on leaves at 96 hpi after trypan blue staining, with the WT showing more infection (bar = 0.5 cm). **(D)** Microscopy of infected areas on leaf surface showing degraded, clumped fungal hyphae on transgenic leaves at 96 hpi (Bar = 0.1 cm).

The complete ORF of *SindOLP* was amplified by PCR using primers NLS1-NLS2 (Supplementary Table S4, Supporting information) containing *Nco*I and *Spe*I site respectively and cloned into pCAMBIA 1302 vector to create pCAMBIA1302-*SindOLP-GFP* (Figure 2C). The fusion construct and the control vector (pCAMBIA1302-GFP) were transformed into *Agrobacterium* LBA4404. Transformation of onion inner epidermal cells was done according to Huang et al. (2011). After 2 days on MS media the transformed cells were visualized under confocal laser scanning microscope (Olympus CLSM, Singapore Model no: 1X81) and images processed with Olympus Fluoview, FV1000.

### Evaluation of response of transgenic sesame to drought and salinity stress

To assay the effect of stress on germination, surface-sterilized seeds from four homozygous transgenic lines (line-3, 7, 10, 12) and WT were germinated in MS-agar medium containing 0, 100, 200 mM NaCl (for salinity stress) and 0, 100, 200 mM mannitol (for drought stress). For each treatment, 12 seeds from WT and different transgenic lines were placed in ½ MS for 7 days at 28 ± 1°C, 16/8 light: dark cycle and their germination frequencies were counted.

For stress on whole plants, WT and transgenic plants were grown in soil-rite in convirons at 70–75% relative humidity, 16/8 light dark cycle, and 28 ± 1°C for 10 weeks with regular watering on every alternate days. Drought stress was simulated by withholding water for 10 days. The plants were then allowed to recover for the next 2 days by watering. Plants were regarded as survivors if there were green, young leaves after the treatment. Survival rate was calculated as the ratio of number of surviving plants over the total number of treated plants. For salinity stress, plants were watered every day with 200 mM NaCl solution for 14 days. Each stress test was done three times.

### Estimation of relative water content (RWC), electrolyte leakage (EL), proline, malonedialdehyde content (lipid peroxidation), phenolics, and flavonoids

RWC was measured according to Hu et al. (2013). Fresh weight (FW) of leaves was recorded followed by soaking the leaves for 4 h in distilled water at room temperature with constant light. The turgid weight (TW) was then recorded. Next the leaves were dried for 24 h at 80°C to obtain the dry weight (DW). RWC was calculated from the equation: RWC (%) = [(FW − DW)/(TW − DW)] × 100.

Electrolyte leakage (EL) was measured according to Hu et al. (2013) with modification. Leaves were cut into strips and incubated in 10 mL distilled water at 28°C for 8 h. Initial conductivity (C1) was measured by a conductivity meter (Systronics, India) followed by boiling the sample in a water bath for 10 min. The leaves were cooled to room temperature electrolyte conductivity (C2) was measured. EL was calculated according to the equation: EL(%) = C1/C2 × 100.

Proline and Malonedialdehyde content were estimated according to Chowdhury et al. (2011). Phenolic content and flavonoids were estimation according to Ray et al. (2015).

### Antioxidant enzyme activity

Antioxidant enzymes viz. Ascobate peroxidase (APX; EC1.11.1.11) and Guaiacol peroxidase (GPX; EC1.11.1.7) were assayed according to Rai et al. (2013).

### Whole plant and detached leaf assay using *Macrophomina phaseolina*

For whole plant infection assay, infected soil treatment according to Kamalkannan et al. (2006) with modifications was utilized. For infected soil preparation, 100 ml of microsclerotial suspension of *M. phaseolina* in sterile water (1 × 10^6^ microsclerotia/ml) was added to 100 g of autoclaved sand-maize medium (19:1 sand and ground maize grain) and allowed to grow for 2 weeks in dark at 28°C. The inoculum was uniformly mixed with 2 kg sterile soil and incubated in dark for 7 days. Ten weeks old plants (WT and transgenic) were replanted in this infected soil, kept in convirons at 28°C, 16/8 h light dark cycle and watered at intervals of 3 days. After 30 days of planting, survival percentage was calculated.

For detached leaf assay, 3 mm discs containing microsclerotia of *M. phaseolina* were scooped out with a sterilized cork-borer and placed in the center of abaxial surface of detached leaves from 10 week old plants. Leaves were kept moist by placing them on wet filter paper inside convirons (temperature 28°C, 16/8 h of light dark cycle). The degree of infection was analyzed by the number of necrotic lesions at 24, 48, 72, 96 hpi and by trypan blue staining (Chowdhury et al., 2014a).

### Oxidative stress experiments and measurement of chlorophyll content

For assaying oxidative damage, healthy leaves from 3 week old seedlings (WT and transgenic lines) were floated on 5 ml of 400 μM H_2_O_2_ for 24 h. For the control set 5 ml deionized water was used. After 24 h these were photographed and the extent of chloroplast damage was observed.

For measurement of chlorophyll content, chlorophyll was extracted from control and treated leaves by 80% acetone and O.D. was measured at 645 and 663 nm using a UV2300II spectrophotometer (Technocomp) according to Chowdhury et al. (2011).

### Assay of H_2_O_2_ and superoxide anion radicals by 3, 3′-diaminobenzidine (DAB) and nitro blue tetrazolium (NBT) staining

Three weeks old seedlings were subjected to stress treatments as follows: for salinity, sprayed with 200 mM NaCl; for drought, sprayed with 200 mM mannitol; for *M. phaseolina* infection, sprayed with microsclerotial suspension (1 × 10^6^ microsclerotia/mL). After stress treatment for 3 days leaves were incubated in DAB (2 mg/mL) and NBT solution (0.1 mg/mL) for 24 h in dark at 28°C then soaked overnight in 95% ethanol to remove chlorophyll (Lu et al., 2013). For the control set, seedlings were sprayed with distilled water and stained as usual.

### Expression analysis of different genes by semi-quantitative RT-PCR and q-RT-PCR

For deciding on which sesame genes to study, five genes were selected (Supplementary Table S3) which are known to function in abiotic and biotic dual stress responses in other plants. These sequences were used to find corresponding genes in sesame from the sesame genome sequence (Zhang et al., 2013). The details of the work are given in Results Section. The list of genes is given in Supplementary Table S3.

Roots of 3 week old plants (lines-3, 7, 10, 12, and WT) were inoculated with *M. phaseolina*. Root were collected at different hours post inoculation (hpi) at the expected peak of expression for each genes viz. for *SiAP2* at 36 hpi, *SiERF* at24 hpi, *SiDef* at 24 hpi, *SiChi* at 48 hpi, and *SiTLP* at 48 hpi. For RT-PCR and qRT-PCR, RNA extraction and cDNA synthesis were done according to published protocol (Chowdhury et al., 2014b). All q-RT-PCR reactions were done following our protocol (Ray et al., 2015) (Applied Biosystems, USA).

Forward and reverse primers of the selected genes (Supplementary Table S4, Supporting information) were designed using Primer-3plus-software (http://www.bioinformatics.nl/cgi-bin/primer3plus/primer3plus.cgi) and IDT-oligo-analyzer (http://www.idtdna.com/analyzer/Applications/OligoAnalyzer/). Amplified genes were sequenced to confirm their identity. Sesame 18S-rRNA and *eIF4*A were used as internal control for RT-PCR and q-RT-PCR respectively (Chowdhury et al., 2014b). The relative abundance of each gene was analyzed using the formula 2^−ΔΔCt^. Three biological replicates were used to ensure the accuracy of results.

### Statistical analyses

Experimental data were subjected to analysis of variance (ANOVA) using software package used for statistical analysis (SPSS version 16, 2007). Significant difference of mean values was compared using Duncan's multiple range test (DMRT) at *P* < 0.05. All the data are the mean ± S.E.M of three independent experiments with three replicates.

## Results

### Molecular characterization of transgenic sesame lines

Thirteen T0 lines having a single copy of *SindOLP*(detected by q-RT-PCR) were selected (Supplementary Figure S1, Supplementary Table S1). After self-pollination, seeds from the T_0_ plants were collected and segregation of the transgene in T1 plants was analyzed by screening in MS medium containing Kanamycin (50 mg/L). Chi-square (*x*^2^) analysis showed 3:1 segregation for the *NptII* gene, indicating Mendelian segregation of single dominant gene in the selected T_1_ lines (Supplementary Table S2). T2 seeds coming from each T_1_ line were separately grouped. Twenty to Twenty-five seedlings of each group were again checked for Kanamycin resistance and for segregation of *SindOLP* sequence through PCR. The group of T2 seedlings that did not show segregation i.e., the group that were all Kanamycin resistant indicated that the mother T1 line was homozygous for the transgene. We selected four such T2 groups namely “line-3,” “line-7,” “line-10,” and “line-12” for molecular and biochemical analysis and stress tolerance assays.

The level of transgene expression in T0 and T2 plants was monitored with “RT-PCR and q-RT-PCR.” In the different T0 lines *SindOLP* expression is similar (Figure 1C). However, in T_2_ plants, there was considerable variation in *SindOLP* expression in the four selected lines (line-3, 7, 10, 12). Highest expression of *SindOLP* was seen in line-7 and lowest in line-12 (Figure 1D). Analysis of tissue specific expression of transgene in line-7 showed ubiquitous expression in different tissues viz. stem, leaf, root, and fruit (Figure 1E).

### Characterization of the protein SindOLP in transgenic sesame lines

Total protein of the four T2 lines (line-3, 7, 10, 12) and WT were analyzed by SDS-PAGE. An extra protein band of ~26 kDa, the expected size of recombinant *SindOLP*, was detected in protein samples of transgenic plants, which was not present in untransformed plants (Supplementary Figure S2A). The proteins were analyzed by western blot using antiserum against purified recombinant *SindOLP*. In western blot this 26 kDa protein band was detected in transgeniclines, but not in the WT (Figure 1F and Supplementary Figure S2B).

### Subcellular localization of SindOLP

To observe the subcellular localization of SindOLP, *SindOLP::GFP* fusion construct, and GFP construct (control) driven by CaMV35S promoter were transiently expressed in the model system for protein localization, the onion epidermal cells (Figure 1G). Control GFP was found to localize in the nucleus and plasma membrane. The recombinant SindOLP*::*GFP was localized primarily in the cytosol, the periphery of the cells, the plasma membrane, at cell boundaries as well as in the nucleus (Figure 1G).

### Overexpression of OLP confers of fungal resistance in transgenic sesame

#### Smaller necrotic lesions in transgenic lines compared to WT

In detached leaf assays, at 96 hpi, symptoms on transgenic leaves consisted of restricted areas of hypersensitive necrotic zones, as opposed to WT leaves which showed extensive areas of necrosis (Figure 2A). The percentage of leaf area affected by necrosis in transgenic lines was significantly less compared to the WT (Figure 2B). Trypan blue staining confirmed less damage of leaf tissues in the transgenic lines compared to WT plants (Figure 2C). Stereo microscopy revealed the presence of damaged fungal hyphae on the leaf surface of transgenic sesame (Figure 2D), indicating the inhibitory role of *SindOLP* in restricting fungal growth, as studied earlier by *in-vitro* assays (Chowdhury et al., 2015).

#### Whole plant bioassays show overexpression of SindOLP leads to enhanced tolerance against *M. phaseolina*

**Figure 3:**
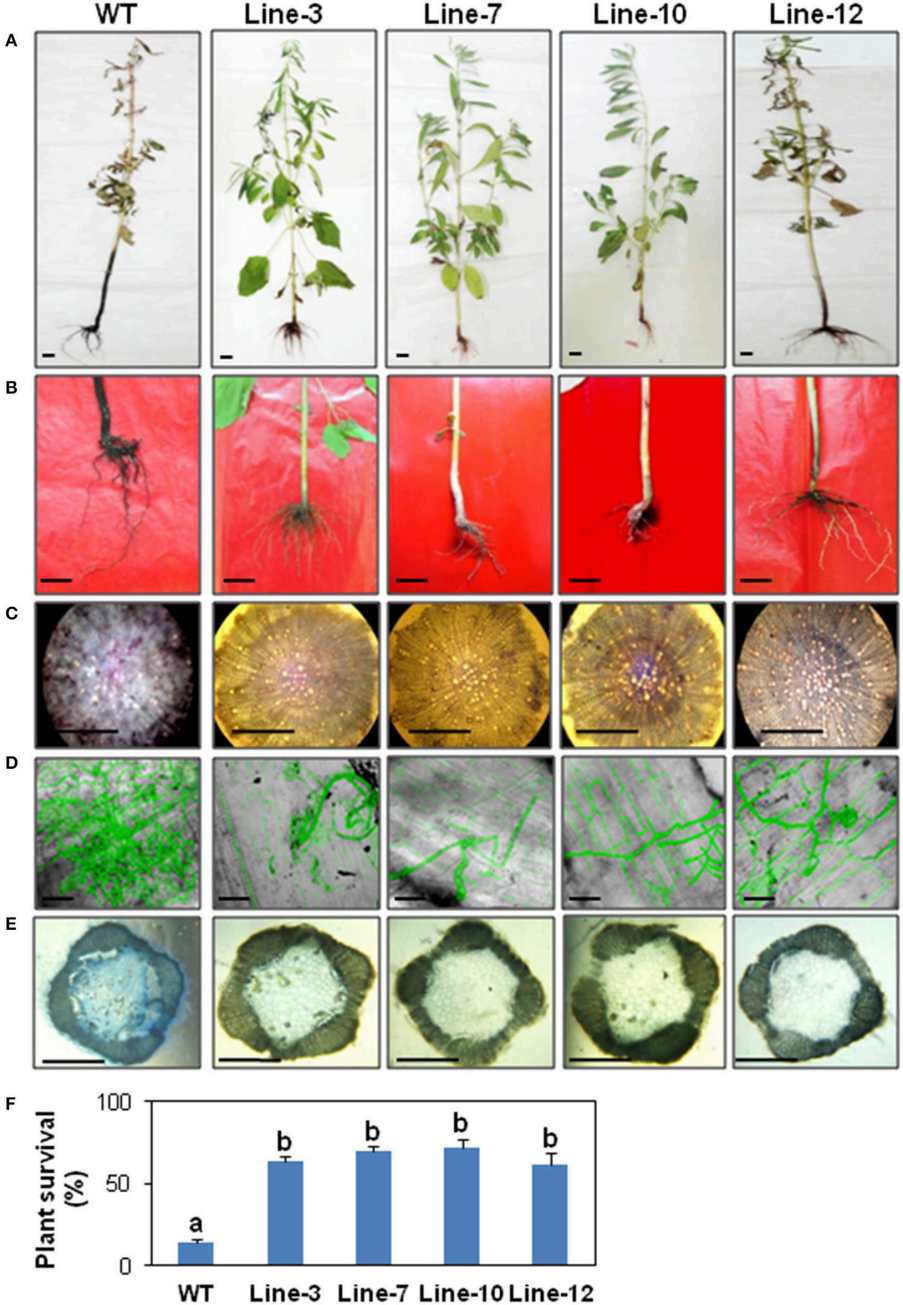
**Whole plant disease assays showing enhanced resistance against *Macrophomina phaseolina* (using normal and GFP transformed pathogen) in transgenic sesame. (A)** Phenotypic appearance of WT and transgenic plants 30 days after infection using infected-soil treatment (bar = 1 cm). **(B)** Symptoms on lower stem and root of WT and transgenic plants (bar = 1 cm). **(C)** TS of roots of WT and transgenic lines with trypan blue staining showing degeneration and necrosis of root tissues in WT but not the transgenic lines. **(D)** Confocal microscopy showing more extensive colonization of GFP-expressing mycelia in root tissue of WT compared to transgenic lines (bar = 200 μm). **(E)** TS of stems showing more stained necrotic tissues in WT compared to transgenic lines. **(F)** Comparison of the survival rate of WT and transgenic lines 1 month after infected-soil treatment (Bar = 0.1 cm). Bars represent mean ± S.E.M of three independent experiments with three replicates. Different letters above bars represent significant difference from WT (*P* < *0.05*).

During whole plant infection assays, within 30 days after planting in infected soil, the WT plants were heavily infected showing typical symptoms of charcoal rot i.e., chlorosis and browning of leaves, wilting of aerial parts, stunted root growth, and dense accumulation of microsclerotia covering the lower stem giving it a charcoal black color. In contrast, the transgenic T2 lines showed less external symptoms (Figures 3A,B). Sections of infected roots and stems of WT and T2 lines revealed fungal colonization with Trypan blue staining and GFP fluorescence. In the WT plants, the roots were heavily colonized with fungal hyphae and in places the xylem vessels were clogged with microsclerotia (Figure 3C). The hyphal growth was found to be impeded in the root tissues of the transgenic plants (Figures 3C,D). Stem sections of WT plants showed signs of rot in the cortical tissue and the conducting vessels (Figure 3E), but no such colonization was seen in stem sections of transgenic plants. The T2 lines showed a higher plant survival (about 60–75%) in comparison to the WT (about 15%; Figure 3F).

#### SindOLP overexpressing sesame lines showed better tolerance to *M. phaseolina* infection estimated by biochemical studies

**Figure 4:**
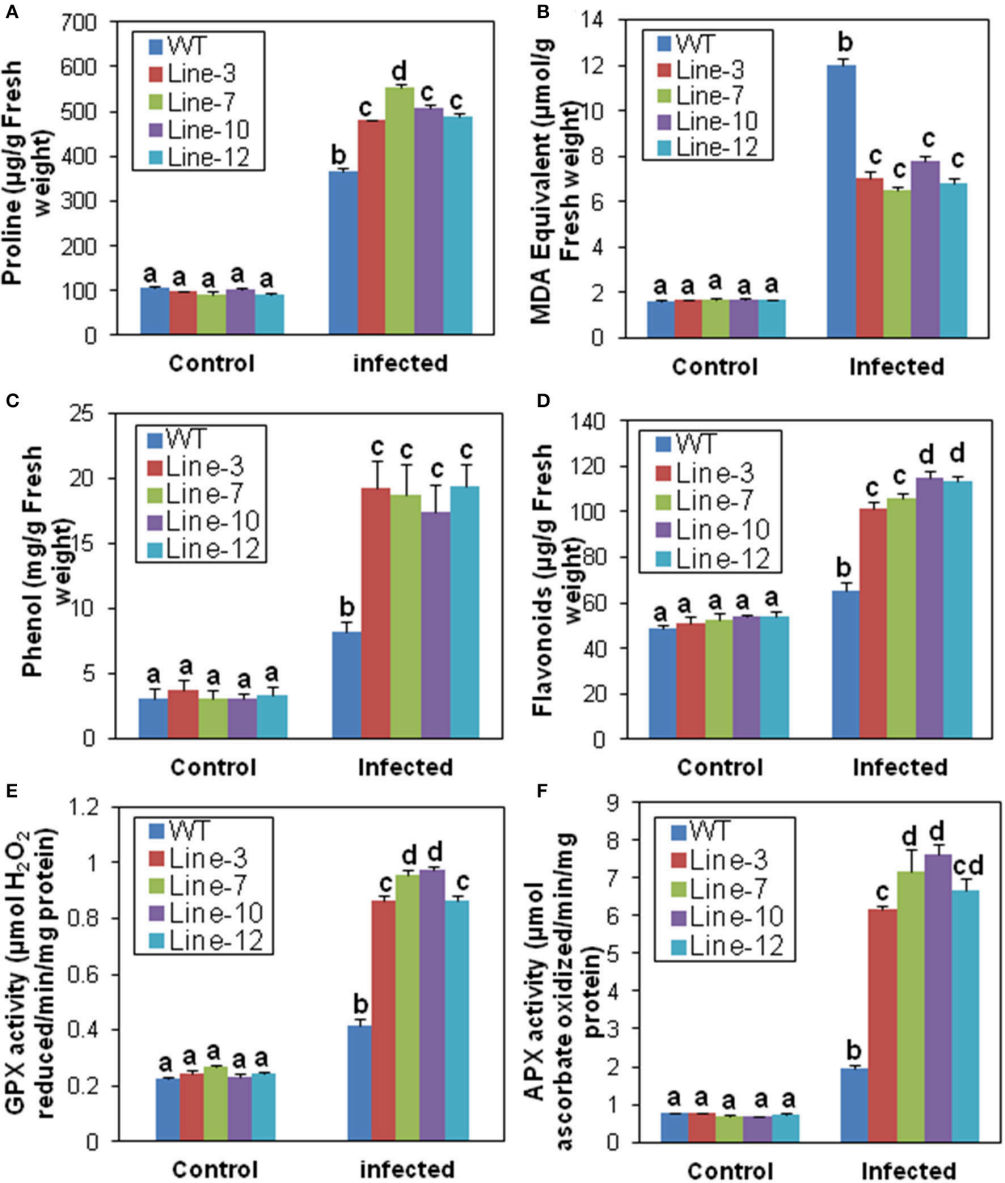
**Biochemical response of *SindOLP* overexpressing sesame plants against *M. phaseolina* infection**. Graphs showing comparison of different biochemical parameters in the WT and transgenic lines.**(A)** Proline **(B)** Lipid peroxidation **(C)** Phenol **(D)** Flavonoids **(E)** Guaiacol peroxidase (GPX) **(F)** Ascorbate peroxidase (APX) activity. Each bar represents mean ± S.E.M of three independent experiments with three replicates. Different letters above bars represent significant difference from WT (*P* < *0.05*).

Under normal conditions, proline levels were similar in the leaf tissues of transgenic and WT plants. Although, the proline content increased after *M. phaseolina* infection in both, it was significantly higher in transgenic plants (480.09–553.01 μg/g) compared to the WT (365.69 μg/g; Figure 4A). Amongst the transgenic lines, line-7 showed maximum increase of proline (Figure 4A). The MDA content, on the other hand, was significantly lower in the transgenic lines (6.504–7.75 μmol/g) than the WT plants (12 μmol/g) after infection (Figure 4B). In the transgenic lines, there were significant increase in total phenolics (17.38–19.34 mg/g) and flavonoids (101.33–114.67 μg/g) in comparison to the WT (8.25 mg/g and 65.46 μg/g, respectively) after infection (Figures 4C,D). Similarly, infection resulted in significant increase in activity of antioxidant scavengers such as GPX and cytosolic APX (total enzyme activity including both of its isoforms) in the transgenic lines compared to their untransformed counter parts (Figures 4E,F). There were no differences in biochemical parameters amongst WT and VC plants after *M. phaseolina* infection (Supplementary Figure S3).

#### Sesame plants overexpressing SindOLP showed altered expression of marker genes functioning in the JA/ET and SA pathways during *M. phaseolina* infection

To understand the mechanism behind the increased stress tolerance, the regulation of five defense-related marker genes functioning in the JA/ET and SA pathways were analyzed. The published sesame genome sequence (Zhang et al., 2013) was used to identify candidate genes that are known to function in other plants in the signal transduction pathways in response to abiotic as well as biotic stress, some of them possibly working as cross-talk factors. To check whether overexpression of *SindOLP* in sesame results in activation of phytohormone defense signaling, transcriptional profiles of the selected genes that function in the two pathways were studied. Supplementary Table S3 shows the list of selected genes and the acronyms we have assigned to them. Supplementary Table S4 shows the list of primers used. The PCR products were sequenced to confirm amplification of the defense gene sequences.

**Figure 5:**
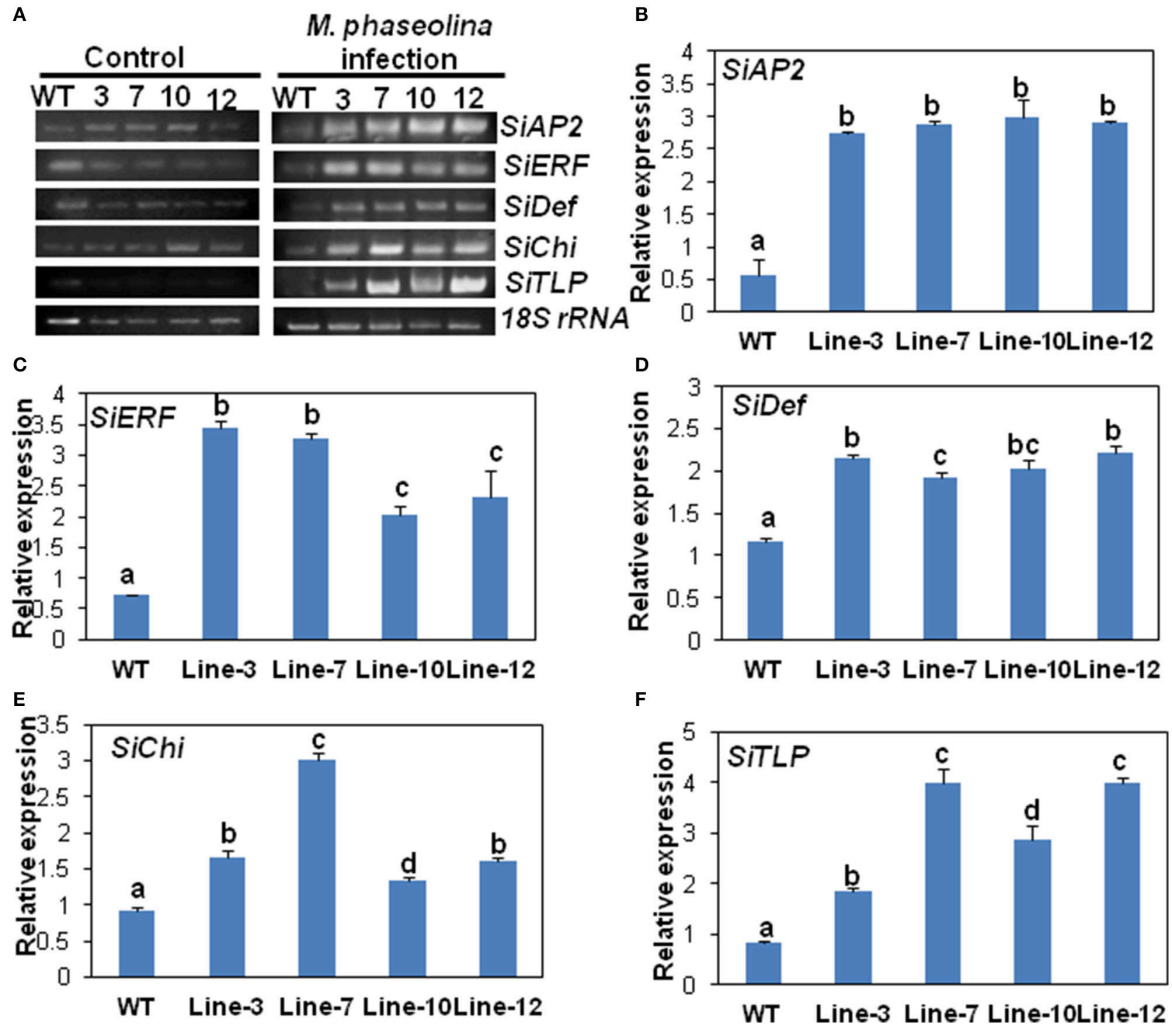
***SindOLP*** **overexpression in sesame leads to up-regulation of defense genes acting in different arms of the signaling cascade upon *M. phaseolina* infection. (A)** RT-PCR showing expression of five defense-related genes in WT and transgenic plants under control conditions and *M. phaseolina* infection. The selected marker genes acting in the JA/ET pathway are *SiAP2* (*Sesamum indicum* EREBP/APETALA 2), *SiERF*(*S. indicum* ethylene-responsive transcription factor ERF071) and *SiDef* (*S. indicum* defensin) and those acting in the SA pathway are *SiChi* (*S. indicum* chitinase-like protein) and *SiTLP* (*S. indicum* thaumatin-like protein). **(B–F)** Graphs showing relative levels of the defense genes in WT and transgenic lines after infection with *M. phaseolina*; *SiAP2* at 36 hpi, *SiERF* at 24 hpi, *SiDef* at 24 hpi, *SiChi* at 48 hpi, and *SiTLP* at 48 hpi. Bars represent mean ± S.E.M of three independent experiments with three replicates. Different letters above bars represent significant difference from WT (*P* < *0.05*).

The expression of the five selected genes in the transgenic lines namely *SiAP2, SiERF, SiDef*, *SiChi, SiTLP* were analyzed by RT-PCR and qRT-PCR. The expressions of these five defense-related genes were similar in WT and transgenic lines prior to *M. phaseolina* infection (Figure 5A). However, after infection with the fungus, expression of the genes was significantly up-regulated in the transgenic lines (Figure 5A). qRT-PCR analyses revealed that there was two- to three-folds increase in the expression of the defense genes in the transgenic lines compared to the WT in response to fungal infection (Figures 5B–F). No significant differences in expression of defense related genes as well as external symptoms were found in WT and VC plants after inoculation with *M. phaseolina* (Supplementary Figure S4).

### Abiotic stress tolerance in the transgenic sesame lines

#### Transgenic sesame overexpressing SindOLP showed salinity and drought tolerance through altered physiological parameters

**Figure 6:**
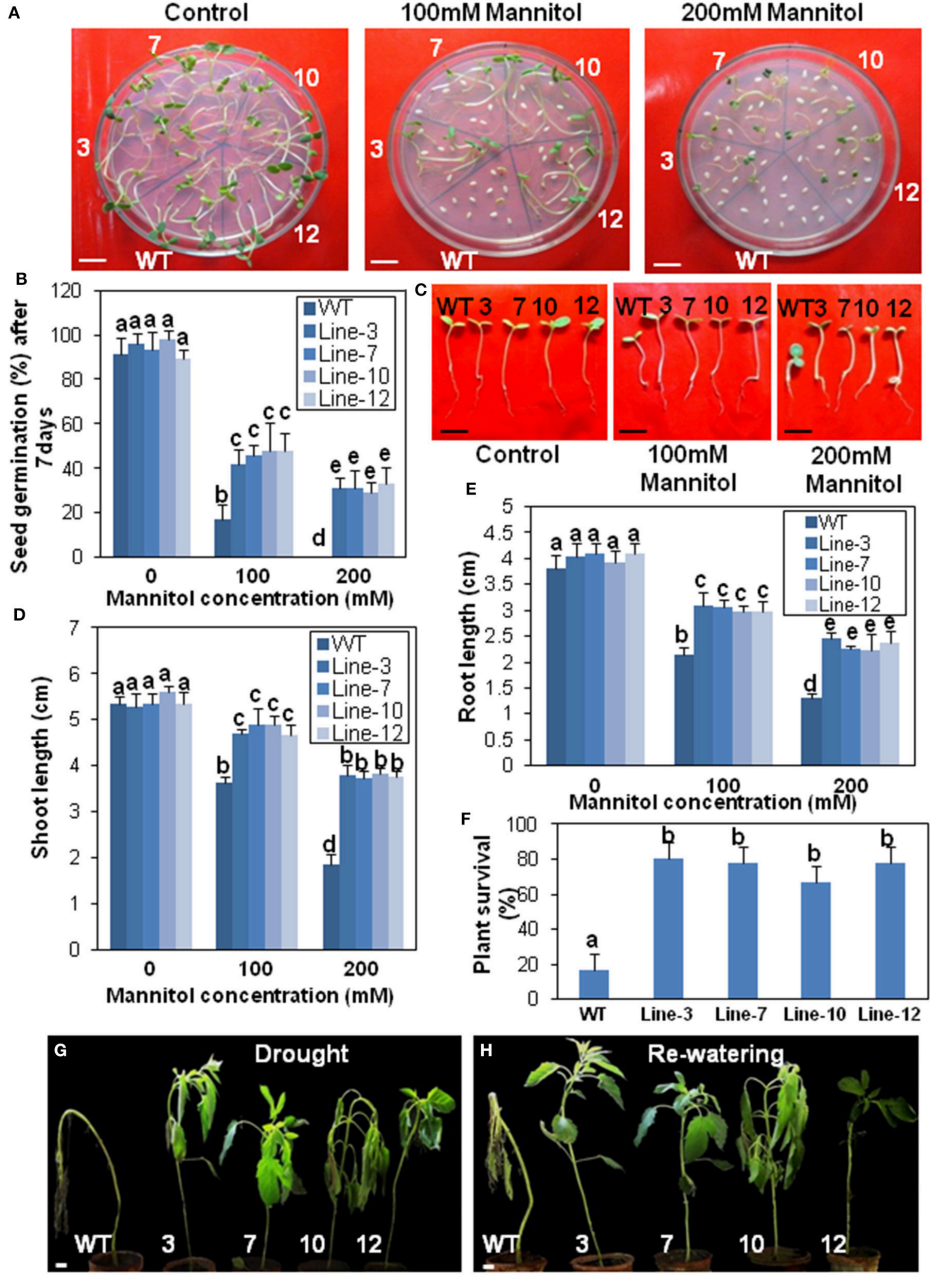
**Transgenic sesame overexpressing *SindOLP* has enhanced drought tolerance. (A)** Germination of transgenic and WT seeds on MS medium with 0, 100, and 200 mM mannitol (bar = 1 cm). **(B)** Graph showing germination frequency of WT and transgenic lines under normal, 100 and 200 mM mannitol treatment. **(C)** Post germination seedling development in the WT and the transgenic lines on MS supplemented with 0, 100, and 200 mM mannitol. The seeds sown on MS medium that showed radicle emergence after 3 days were transferred to MS medium containing different concentrations of mannitol. Photographs were taken 7 days after transfer (bar = 1 cm) **(D,E)** Primary shoot length and root length of seedlings 10 days after germination in different mannitol concentrations. **(F)** Survival rates of 10 weeks old plants where drought conditions were simulated by withholding water for 10 days following 2 days of recovery by re-watering. Bars represent mean ± S.E.M of three independent experiments with three replicates. Different letters above bars represent result significantly different from WT at *P* < *0.05*. **(G,H)** Representative photographs of 10 weeks old WT and transgeniclines grown under drought conditions for 10 days then watered for 2 days to allow for recovery (bar = 1 cm).

**Figure 7:**
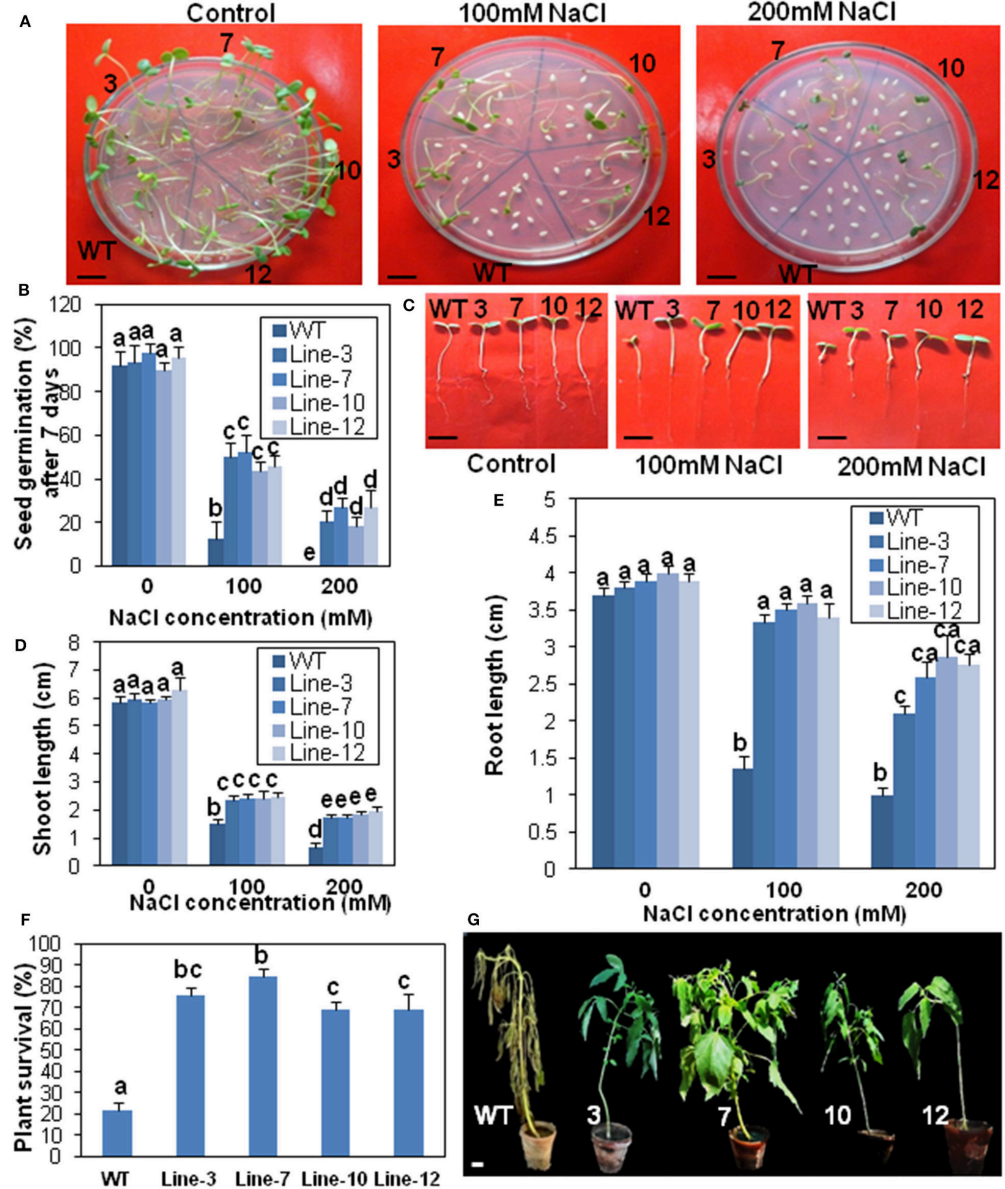
**Transgenic sesame overexpressing *SindOLP* has enhanced salinity tolerance. (A)** Germination of transgenic and WT seeds on MS medium with 0, 100, and 200 mM NaCl (bar = 1 cm). **(B)** Graph showing germination frequency of WT and transgenic lines under normal, 100 and 200 mM NaCl treatment. **(C)** Post-germination seedling development of the WT and the transgenic lines on MS supplemented with 0, 100, and 200 mM NaCl. The seeds sown on MS medium that showed radicle emergence after 3 days were transferred to MS medium containing different concentrations of NaCl. Photographs were taken 7 days after transfer (bar = 1 cm). **(D,E)** Primary shoot and root length of seedlings 10 days after germination in different concentrations of NaCl. **(F)** Survival rates of 10 weeks old plants watered with 200 mM NaCl solution for 14 days. Bars represent mean ± S.E.M of three independent experiments each with three replicates. Different letters above bars represent significant difference from WT at *P* < *0.05*. **(G)** Representative photographs of 10 weeks old WT and transgeniclines under salt stress for 14 days (bar = 1 cm).

The seeds of the transgenic lines were assayed for drought and salinity tolerance (Figures 6, 7). Under drought and salinity treatments, germination frequency of seeds was reduced in all seeds, but the transgenic seeds fared better than the WT (Figures 6A, 7A). In 100 mM mannitol (drought stress), seed germination frequency in the WT was significantly lower (16.62%) than that of the transgenic lines which ranged from 41.66 to 47.91%. Similarly in 100 mM NaCl (salinity stress), transgenic lines had higher seed germination frequency (43.74–52.08%) in comparison to the WT (12.5%). At the highest drought (200 mM mannitol) and salinity (200 mM NaCl) conditions, seed germination was completely blocked for WT plants. However, some seeds from the transgenic lines could tolerate this extreme stress. These had a germination frequency of 29.16–33.33% in drought stress (Figure 6B) and 18.7–27.08% in salinity stress (Figure 7B).

Similarly, there was a significant reduction in root- and shoot-length in response to drought and salinity stress, in the case of WT plants compared to the transgenic lines at different stress levels (Figures 6C–E, 7C–E).

In survival experiments, most of the 10 weeks old WT plants after 10 days of drought treatment were irreversibly wilted but the transgenic plants were less affected (Figure 6F). Around 80% of transgenic plants recovered during re-watering compared to only 20% of the WT, indicating better sustenance of the transgenic lines under drought conditions (Figures 6G,H).

For survival assay under salinity stress, only 22% WT plants survived 14 days of salinity stress, whereas transgenic plants showed significantly higher survival (68–84%; Figure 7F). After 2 weeks of treatment, WT plants showed severe chlorosis and wilting but the transgenic plants sustained without visible signs of stress (Figure 7G).

Relative water content (RWC), an evaluation of plant water status, was higher in the transgenic lines than WT after 10 days drought stress. After re-watering, RWC increased in WT plants, but remained lower compared to the re-watered transgenic plants (Supplementary Figure S5A). Transgenic plants coped with water loss through well-developed root system with longer and thicker roots compared to WT plants (Supplementary Figures S5B,C). Electrolyte leakage, an important indicator of membrane damage, was significantly higher in WT plants than transgenic lines, suggesting less membrane damage during drought and salinity stress in the transgenic lines (Supplementary Figure S5D).

Since stomata often close in order to limit water loss by transpiration during abiotic stress like drought or salinity, stomatal apertures of the transgenic, and WT plants were compared before and after drought and salinity stress. The aperture of the open stomata was similar in the WT and transgenic plants before stress. However, during drought and salinity stress the *SindOLP* overexpressing plants exhibited a smaller stomatal aperture than the WT plants (Supplementary Figures S5E,F).

#### Transgenic sesame over-expressing OLP showed enhanced drought and salinity tolerance through altered biochemical parameters

The proline accumulation in transgenic lines was significantly higher than WT plants after drought and salinity stress (Supplementary Figure S6A). The Malonedialdehyde (MDA) content of WT and the transgenic lines increased after drought and salinity stress in comparison to control. However, the MDA content of transgenic lines were significantly lower than that of WT (Supplementary Figure S6B) indicating less membrane injury in the transgenic plants. After stress, transgenic lines accumulated significantly higher phenolics and flavonoids than WT (Supplementary Figures S6C,D). Moreover, the transgenic lines showed significantly higher GPX and cytosolic APX activities than WT plants under stress (Supplementary Figures S6E,F). There were no differences amongst WT and VC (vector control) plants after abiotic stresses (Supplementary Figure S3).

#### SindOLP overexpression induces oxidative stress tolerance in sesame

**Figure 8:**
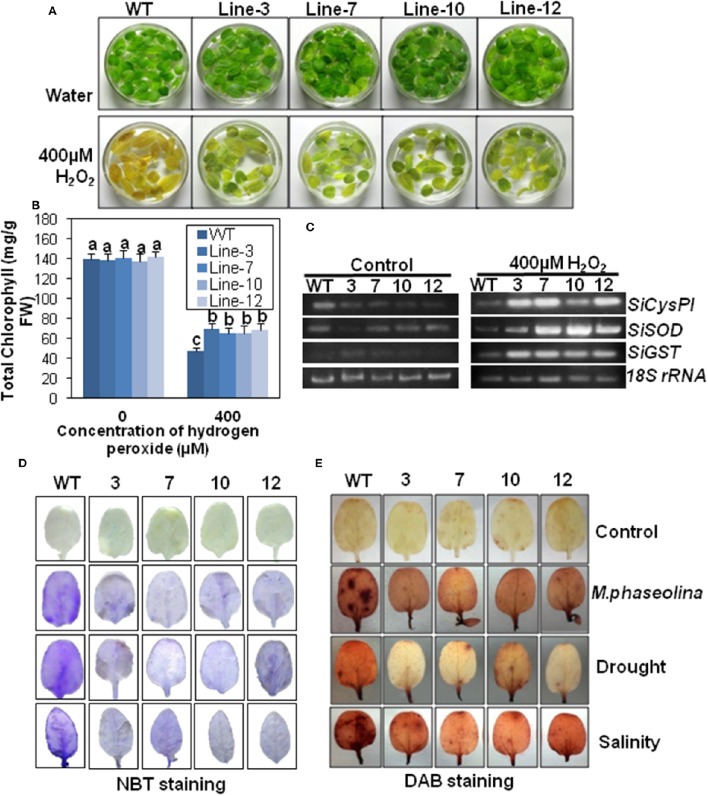
***SindOLP*** **overexpression results in oxidative stress tolerance and reduced ROS accumulation in transgenic sesame in response to abiotic and biotic stress. (A)** Leaves from WT and transgenic lines incubated in 400 μM H_2_O_2_ and water as control at 24 h showing transgenic leaves remained green while WT leaves turned yellow (bar = 2 cm). **(B)** Graph showing chlorophyll content of leaves of WT and transgenic lines after 24 h of incubation in 400 μM H_2_O_2_. Each bar is mean ± S.E.M of three independent experiments with three replicates. Different letters above bars represent significant differences from WT (*P* < *0.05*). **(C)** RT-PCR showing expression of genes coding for antioxidants like *SiCysPI* (*Sesamum indicum* cysteine proteinase inhibitor), *SiSOD* (*S. indicum* Superoxide dismutase), and *SiGST* (*S. indicum* glutathione-S-transferase) in WT and transgenic lines under control and 400 μM H_2_O_2_ treatment. **(D,E)** Abiotic and biotic stress induced accumulation of O2^−^, H_2_O_2_ in WT, and transgenic plants, detected by NBT **(D)** and DAB staining **(E)** (bar = 0.5 cm).

The leaves of WT plants changed from green to yellow after H_2_O_2_-induced oxidative stress for 24 h, but the leaves from transgenic plants remained green (Figure 8A). The transgenic leaves had higher chlorophyll content (65.93–70.1 mg/g) than leaves from WT plants (47.41 mg/g) post oxidative stress (Figure 8B). There was no significant difference in chlorophyll content between the WT and VC plants before and after stress (Supplementary Figure S7).

#### Transgenic lines showed increased transcript levels of three enzymes involved in ROS signaling

To understand the mechanism behind the increased oxidative stress tolerance, the expression of genes involved in ROS signaling viz. superoxide dismutase (*SiSOD*), cysteine protease inhibitor (*SiCysPI*), glutathione-S-transferase (*SiGST*; Supplementary Tables S3, S4) was observed. Under control conditions, there was no difference in the expression levels of *SiSOD, SiCysPI*, and *SiGST* between the WT and the transgenic plants. After 400 mM H_2_O_2_ treatment for 24 h, there was higher accumulation of *SiCysPI, SiSOD*, and *SiGST* transcripts in transgenic plants than WT (Figure 8C).

#### Transgenic plants overexpressing SindOLP showed reduced ROS accumulation

Under normal conditions, the leaves of WT and transgenic lines showed no NBT staining. After stress treatment the leaves of plants overexpressing *SindOLP* exhibited less blue staining than WT plants, indicating less accumulation of ${\text{O}}_{2}^{-}$ (Figure 8D). Likewise, H_2_O_2_ contents of the WT and transgenic plants were similar under normal conditions. After infliction of biotic and abiotic stresses, the WT plants took a deeper DAB (3, 3′-Diaminobenzidine) stain compared to the transgenic lines indicating that the accumulation of H_2_O_2_ was considerably higher in the WT than the transgenic plants (Figure 8E).

## Discussion

Plants, unlike animals cannot move away from the sources of stress (Huey et al., 2002). At any given time, each of the different parts of a plant is exposed to several abiotic and biotic stresses. How plants adjust their responses to each of the stresses simultaneously and yet coordinately is an interesting area of research.

The overexpression of *SindOLP*, cloned in this laboratory (Chowdhury et al., 2015), in sesame presented a feasible approach to not only generating multi-stress tolerant crop lines, but also to provide us with the opportunity to study plant response to abiotic and biotic stresses. This study is the first report of transgenic sesame and also the first study involving gene regulation in sesame. Moreover, we get an insight into the molecular mechanism as to how OLPs enhance multi-stress tolerance, which hitherto has not been investigated.

The transgenic sesame lines overexpressing *SindOLP* showed resistance against the abiotic stresses of drought and salinity and the biotic stress caused by a fungal pathogen. The transgenic lines showed better seed germination, root/shoot length, RWC, survival rate, and sustenance under drought/salinity stress. At the cellular level, salinity and drought stresses is known to cause accumulation of MDA, proline, and secondary metabolites like flavonoids and phenolics (Tang et al., 2014). In this study lower MDA and lipid peroxidation, higher accumulation of proline, secondary metabolites, and improved antioxidant enzyme activities in the transgenic lines accounted for enhanced tolerance against drought, salinity, and pathogen. Less electrolyte leakage in transgenic lines during stress, indicate less membrane injury in the cells than WT plants. Subcellular localization experiments showed that the *SindOLP::GFP* fusion protein localized predominantly in the cytosol and plasma membrane which is consistent with previous reports about a pepper osmotin (Choi et al., 2013) and the thaumatin-like proteins (Kim et al., 2009).

Stomatal aperture is an important parameter in drought tolerance. The present study shows smaller stomatal apertures in the transgenic lines than WT leaves, presumably in an attempt to reduce water loss through transpiration and maintaining cellular osmolarity. Previous reports show that drought and salt tolerance is mediated through the control of stomatal aperture in rice (Huang et al., 2009) and maize (Cai et al., 2014).

Biotic and abiotic stresses commonly impinge on the redox status (Suzuki et al., 2014) and increase in activities of antioxidative enzymes is closely related to salt, drought, and pathogen resistance (Tamirisia et al., 2014). In this study the overexpression of *SindOLP* resulted in higher antioxidant activities in the transgenic lines. Although, incubation of leaves for DAB and NBT staining is stress itself, still less H_2_O_2_ and superoxide accumulation in transgenic lines compared to WT, under biotic/abiotic stress treatments speaks of less ROS accumulation as consequence of SindOLP overexpression. To elucidate the molecular mechanism behind the antioxidant role of *SindOLP*, we investigated the regulation of genes coding for ROS-scavenging enzymes in the transgenic lines. We found that *SindOLP* overexpression resulted in up-regulation of the ROS-scavenging enzyme genes *viz. SiCysPI, SiSOD*, and *SiGST*, during oxidative stress. Therefore, these results indicate that *SindOLP* takes part in the regulation of the ROS scavenging pathway.

Although, different in nature, salt, drought, and cold stresses are known to activate common sets of genes in plants (Tamirisia et al., 2014). Recent studies indicate that biotic stress also plays into the common network of defense signaling. The common nodal point from which the abiotic and biotic stress signaling pathways diverge, is around ROS production which in turn affect the two arms of phytohormone defense signaling viz. JA/ET and SA pathways (Kissoudis et al., 2014; Rejeb et al., 2014). In this study, to get an insight into the molecular mechanism behind the role of *SindOLP* in conferring resistance to abiotic and biotic stresses, we had selected marker genes that take part in the JA/ET and the SA signaling arms. The *AP2, ERF*, and *Defensin* are marker genes for the JA/ET pathway while *Chi* and *TLP* are markers for SA signaling in plants. Expression analyses of these genes in sesame indicated that *SindOLP*-dependent activation of these defense genes plays a key role in enhanced abiotic/biotic stress tolerance in the transgenic plants, which is mediated through SA as well as JA/ET signaling pathways. Since OLPs are induced by SA and JA (Jami et al., 2007), the up-regulation of defense-related genes in transgenic sesame in response to fungal infection was due to the evocation of phytohormone defense signaling as a direct consequence of the constitutive overexpression of *SindOLP*. Although, the complex regulatory mechanism behind biotic/abiotic stress signaling involving *SindOLP* cannot be commented upon, this study indicate that it functions through integrated activation of several components of the stress signaling cascade.

On the whole it can be said that the overexpression of *SindOLP* in sesame led to improved drought, salinity, oxidative stress and disease tolerance through changes in some physiological, biochemical parameters and an integrated effect on the regulation of stress-responsive genes. Although, the complex regulatory mechanism involving *SindOLP* is not fully understood, our results give us an insight at the fundamental level, showing that the *SindOLP* gene has explicit functional role in abiotic/biotic stress signaling involving multiple components.

## Author contributions

SK planned the project, drafted the experiments and acquired funding. SC and AB performed all the experiments and analyzed data. SC and SK wrote the manuscript. All authors read and approved the final version of the manuscript.

### Conflict of interest statement

The authors declare that the research was conducted in the absence of any commercial or financial relationships that could be construed as a potential conflict of interest.
