# Supplementary material for: Overexpression of a New Osmotin-Like Protein Gene (SindOLP) Confers Tolerance against Biotic and Abiotic Stresses in Sesame
Source: Front Plant Sci. 2017 Mar 28;8:410. doi: 10.3389/fpls.2017.00410 (PMC5368222; doi:10.3389/fpls.2017.00410)
Supplement: Supplementary file 1 [file DataSheet1.docx]

**Supplementary materials**

**Supplementary Table S1.Copy number of *SindOLP* in different T0 transgenic lines as estimated by qPCR**

| **T0-transgenic lines** | **Copy number of *SindOLP* in transgenic lines** |
| --- | --- |
| T0-1 | 1 |
| T0-2 | 1 |
| T0-3 | 1 |
| T0-4 | 1 |
| T0-5 | 1 |
| T0-6 | 1 |
| T0-7 | 1 |
| T0-8 | 1 |
| T0-9 | 1 |
| T0-10 | 1 |
| T0-11 | 1 |
| T0-12 | 1 |
| T0-13 | 1 |

**SupplementaryTable S2.Segregation of Kanamycin resistance trait in T1 generation of transgenic sesame.**

| **T0 line** | **Number of T1 lines tested for Kanamycin resistance** | **Kanamycin resistant lines (%)** | **Kanamycin sensitive lines (%)** | **χ^2^ value*** | **Ratio of resistant: susceptible seedlings** |
| --- | --- | --- | --- | --- | --- |
| T0-1 | 23 | 19(82.60) | 4(17.39) | 0.7101 | 3:1 |
| T0-2 | 20 | 16(80) | 4(20) | 0.266 | 3:1 |
| T0-3 | 18 | 14(77.77) | 4(22.22) | 0.0735 | 3:1 |
| T0-4 | 24 | 19(79.16) | 5(20.83) | 0.2216 | 3:1 |
| T0-5 | 17 | 13(76.47) | 4(23.52) | 0.01960 | 3:1 |
| T0-6 | 19 | 14(73.68) | 5(23.61) | 0.0174 | 3:1 |
| T0-7 | 21 | 15(71.42) | 6(28.57) | 0.1428 | 3:1 |
| T0-8 | 20 | 14(70) | 6(30) | 0.266 | 3:1 |
| T0-9 | 22 | 18(81.81) | 4(18.18) | 0.5453 | 3:1 |
| T0-10 | 24 | 17(70.83) | 7(29.16) | 0.2221 | 3:1 |
| T0-11 | 23 | 18(78.26) | 5(21.73) | 0.1304 | 3:1 |
| T0-12 | 25 | 19(76) | 6(24) | 0.0133 | 3:1 |
| T0-13 | 21 | 16(76.19) | 5(23.80) | 00158 | 3:1 |

*P<0.05 (significantly different at this level)

**Supplementary Table S3. List of genes studied in the transgenic and WT sesame under biotic and abiotic stress.**

| **Acronym of gene used in present study** | **Function of gene** | **Genbank accession number** |
| --- | --- | --- |
| ***SiAP2*** | *Sesamum indicum* EREBP/AP2 (Apetala 2) | KM190074.1 |
| ***SiERF*** | *Sesamum indicum* ethylene-responsive transcription factor ERF071 | [XM_011081322.1](http://www.ncbi.nlm.nih.gov/nucleotide/747065923?report=genbank&log$=nuclalign&blast_rank=1&RID=CM4NERV601R) |
| ***SiDef*** | *Sesamum indicum* defensin | [XM_011085787.1](http://www.ncbi.nlm.nih.gov/nucleotide/747074213?report=genbank&log$=nucltop&blast_rank=1&RID=CM5B6UJG014) |
| ***SiChi*** | *Sesamum indicum* chitinase like protein | XM_011075613.1 |
| ***SiTLP*** | *Sesamum indicum* thaumatin like protein | [XM_011085956.1](http://www.ncbi.nlm.nih.gov/nucleotide/747043531?report=genbank&log$=nucltop&blast_rank=1&RID=CM6ASCB8014) |
| ***SiSOD*** | *Sesamum indicum* Superoxide dismutase (Cu-Zn) | [XM_011092562.1](http://www.ncbi.nlm.nih.gov/nucleotide/747086705?report=genbank&log$=nuclalign&blast_rank=1&RID=CM4JPKBF01R) |
| ***SiGST*** | *Sesamum indicum* glutathione S-transferase | [XM_011081394.1](http://www.ncbi.nlm.nih.gov/nucleotide/747066055?report=genbank&log$=nuclalign&blast_rank=1&RID=CM4X27Z7014) |
| ***SiCysPI*** | *Sesamum indicum* cysteine proteinase inhibitor A | [XM_011082189.1](http://www.ncbi.nlm.nih.gov/nucleotide/747067530?report=genbank&log$=nuclalign&blast_rank=1&RID=CM63ZZES015) |

**Supplementary Table S4. List of primers used in this study.**

| **Gene name** | **Forward primer(5’-3’)** | **Reverse primer(5’-3’)** | **Size of amplicon** |
| --- | --- | --- | --- |
| ***SiAP2*** | AGGGTTTAGAGAGCACCACC | CTCTGCGCTGCTTCGTTATT | 192 |
| ***SiERF*** | AAACTTCCCAAAGCACTTCC | ACGAAAGTCAACGAGGTTAC | 200 |
| ***SiDef*** | TCTTTTGTCATCGTTCCGCG | CAACGAAGACGTGGTTTGTG | 214 |
| ***SiChi*** | TTTGGGTACAACTTTAAGCG | GTTGGTCTGCTAAAGCCTAA | 187 |
| ***SiTLP*** | CGTCATAGACCCCCTCAACT | AATCGCGTAAACGAGGAAAA | 202 |
| ***SiSOD*** | TGTACTTTCGGTACCTCAGG | TCTACCTAGTCGGGTGAAAC | 172 |
| ***SiGST*** | TAGAAGTTTCGGACCCTGGA | ACTACGACAACCAGTCTTCG | 183 |
| ***SiCysPI*** | AAACACCATCAGTAGCTGC | CACATACTCCGGTTCCACAC | 151 |
| ***NLS*** | CGC**CCATGG**TGGGCTACTTGAGATCT  ***NcoI*** | CCC**ACTAGT**TTACTTGGCCACTTCATC  ***SpeI*** | 744 |
| ***SindOLP*** | CGCGCTGCGACTATCGAGGTACGC | CCCACCCTTAGGACAAAAGACAACCC | 730 |
| ***eIF4A*** | AGCCCGTCCGCATTCT | AAGCCAGTCAACCTTTCTCC | 176 |
| ***18S-rRNA*** | TTCCATGCTAATGTATTCAGAG | ATGGTGGTGACGGGTGAC | 500 |

**Supplemental Figure S1. SindOLP in transgenic sesame lines.**(A) Detection of copy number of SindOLP in transgenic sesame lines by q-RT-PCR.(B) Sequence of SindOLP.

**Supplemental Figure S2. Detection of expressed SindOLP in transformed sesame lines by SDS-PAGE and immunoblot.** (A) Total soluble proteins from different transgenic lines were analyzed by SDS-PAGE. M= molecular weight marker, 1= WT nontransgenic plant (negative control), 2= line-3, 3= line-7, 4= line-10, 4= line-12, 5= *SindOLP* overexpressed in *E.coli* (positive control). (B) Immunoblot analysis of WT and transgenic sesame lines with anti-SindOLP antibody, Lane1= WT plant, Lane2= line-3, Lane3= line-7, Lane4= line-10, Lane5= line-12.

**Supplemental Figure S3. Biochemical response of WT and transformed vector control (VC) sesame plants against drought, salinity and infection with *M. phaseolina.*(A)** Proline content **(B)** Lipid peroxidation **(C)** Phenol **(D)** Flavonoids **(E)** Ascorbate peroxidase (APX) activity **(F)** Guaiacol peroxidase (GPX) activity. Bars represent mean ± S.E.M of three independent experiments with three replicates. Bars with similar letters are not significantly different (*P<0.05*).

**Supplemental Figure S4. Expression analyses of five defense related genes in WT and transformed vector control (VC) sesame plants in response to *M phaseolina* infection.**The timepoints were selected based on the expected peak expression time of each gene. **(A)**External appearance of WT and transgenic plants at 30 days after infection using infected soil treatment. **(B)***SiAP2* at 36 hpi,**(C)***SiERF* at24 hpi, **(D)***SiDEF* at24 hpi **(E)***SiTLP* 48 hpi **(F)***SiCHI* at 48 hpi *.* Each bar represents mean ± S.E.M of three independent experiments with three replicates. Bars sharing similar letters are not significantly different (*P<0.05*).

**Supplemental Figure S5. Overexpression of *SindOLP* enhanced drought and salinity tolerance in sesame through modification of several physiological parameters. (A)** Relative water content (RWC) in WT and transgenic lines under normal, drought and re-watering period. **(B, C)**Phenotype of WT and transgenic roots under drought stress with transgenic lines showing longer roots.**(D)** Electrolyte leakage in WT and transgenic lines upon treatment with 200mM mannitol, 200mM NaCl. **(E)** Stomatal apertures in WT and transgenic lines before and after drought/salinity stress with transgenic lines showing closing of stomata in response to stress (Bar=200µm). **(F)** Graph showing differences in stomatal aperture during drought and salinity stress in WT and transgenic lines. Bars represent mean±S.E.M of three independent experiments with three replicates. Different letters above bars represent significant difference from WT (*P<0.05*).

**Supplemental Figure S6. Biochemical responses of *SindOLP* overexpressing sesame against drought and salinity stress.(A)** Proline **(B)** Lipid peroxidation **(C)** Phenol **(D)** Flavonoids **(E)** Guaiacol peroxidase (GPX) **(F)** Ascorbate peroxidase (APX) activity. Bars represent mean±S.E.M of three independent experiments with three replicates. Different letters above bars represent significant difference from WT (*P<0.05*).

**Supplemental Figure S7. Evaluation of responses of WT and transformed vector control (VC) sesame plants under oxidative stress*.*(A)** Comparison of leaves from WT and VC plants under oxidative stress (incubation in 400mM H_2_O_2_) and water (control) for 24hrs. **(B)** Graph showing chlorophyll content of leaves of WT and VC plants after 24hrs of incubation in 400mM H_2_O_2._ Bars represent mean ± S.E.M of three independent experiments with three replicates. Bars having similar letters are not significantly different (*P<0.05*).

**SupplementalFigure S8.*Agrobacterium* mediated transformation of the charcoal rot pathogen *Macrophomina phaseolina* using binary vector pCAMBIA 1302.(A)** Schematic diagram of the T-DNA part of the vector. **(B)** Transformed *M. phaseolina* colony showing GFP fluoresce under UV-light. **(C)** Transformed hyphae of *M. phaseolina* expressing GFP seen under confocal microscope (bar= 100µm). **(D)** Brightfield image of GFP expressing fungal hyphae on sesame roots (bar= 100µm), **(E)** the same frame under UV (bar= 100µm).
