# Supplementary figures and images for: Overexpression of a New Osmotin-Like Protein Gene (SindOLP) Confers Tolerance against Biotic and Abiotic Stresses in Sesame
Source: Front Plant Sci. 2017 Mar 28;8:410. doi: 10.3389/fpls.2017.00410 (PMC5368222; doi:10.3389/fpls.2017.00410)

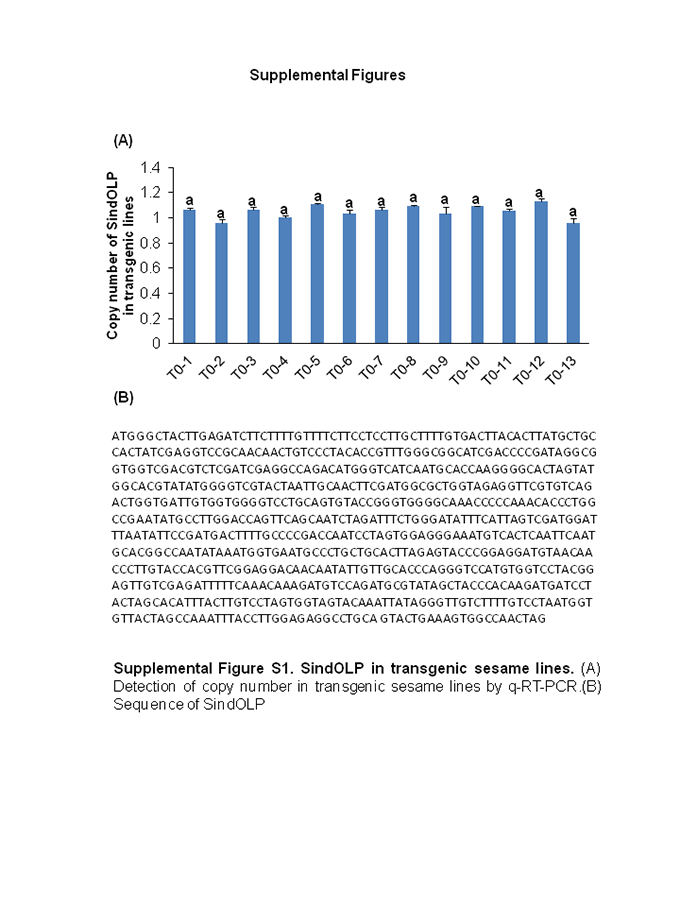

Supplement: Supplementary file 2 [file Image1.TIF]

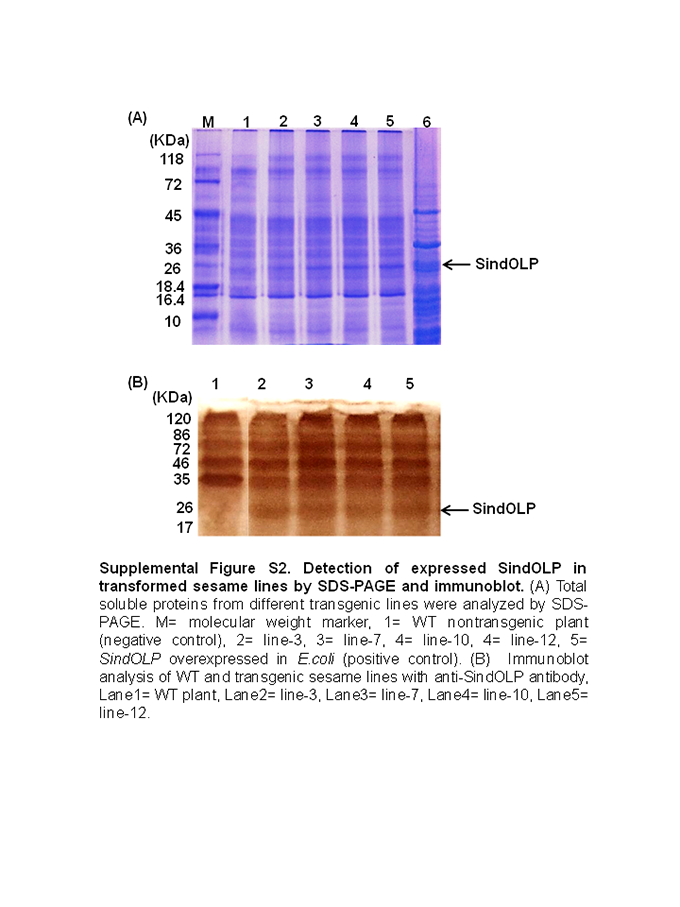

Supplement: Supplementary file 3 [file Image2.TIF]

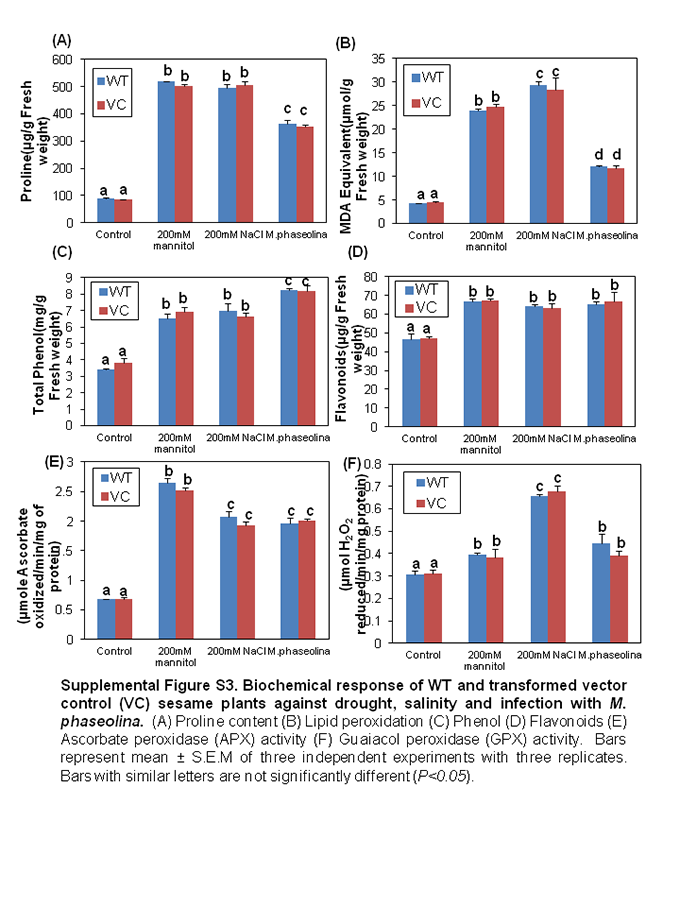

Supplement: Supplementary file 4 [file Image3.TIF]

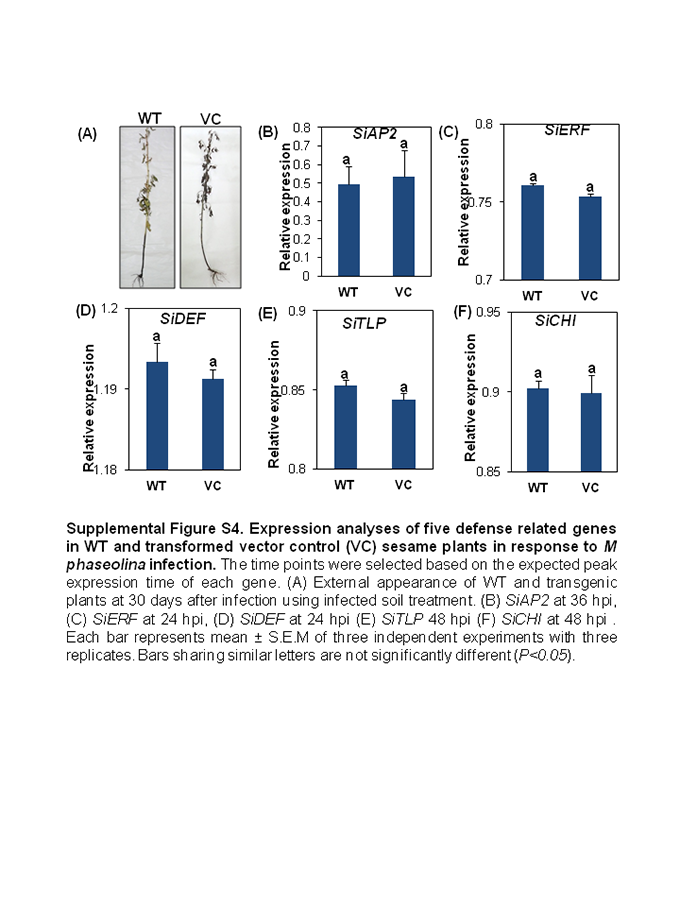

Supplement: Supplementary file 5 [file Image4.TIF]

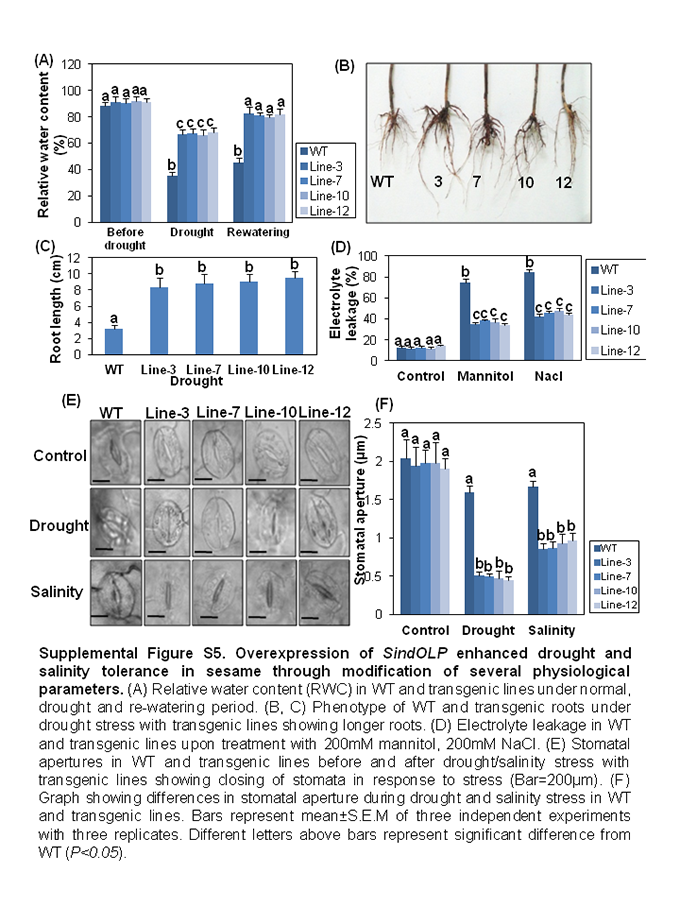

Supplement: Supplementary file 6 [file Image5.TIF]

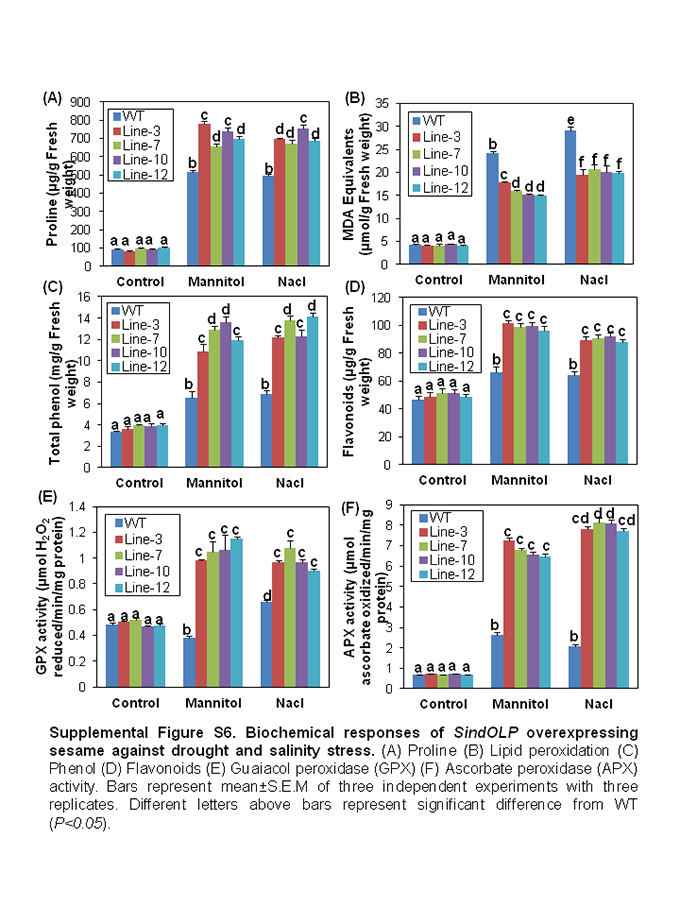

Supplement: Supplementary file 7 [file Image6.TIF]

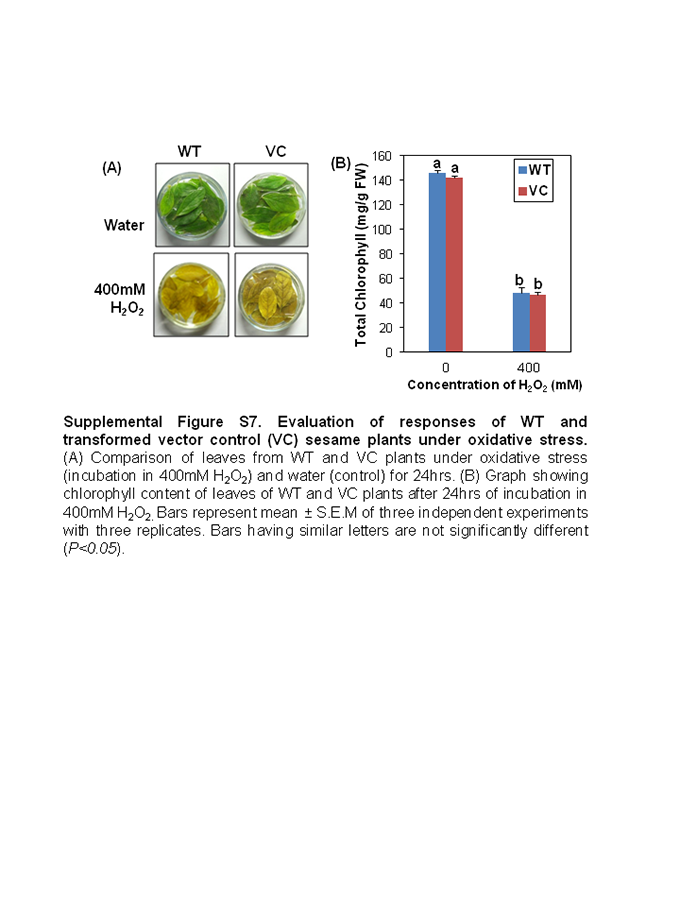

Supplement: Supplementary file 8 [file Image7.TIF]

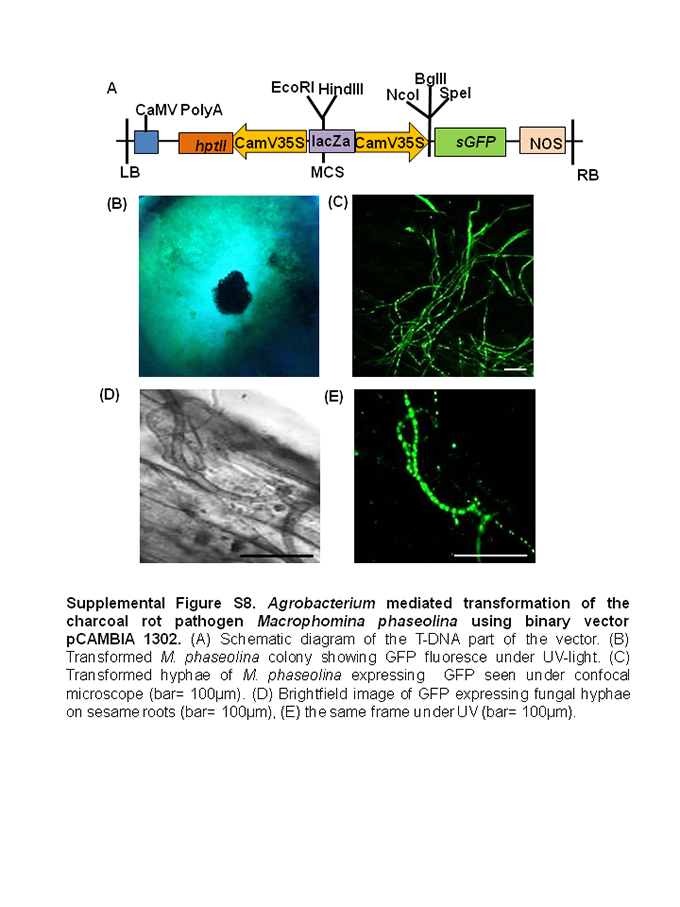

Supplement: Supplementary file 9 [file Image8.TIF]
